# miRNAs in Health and Disease: A Focus on the Breast Cancer Metastatic Cascade towards the Brain

**DOI:** 10.3390/cells9081790

**Published:** 2020-07-28

**Authors:** Marta Sereno, Mafalda Videira, Imola Wilhelm, István A. Krizbai, Maria Alexandra Brito

**Affiliations:** 1 Research Institute for Medicines (iMed.ULisboa), Faculdade de Farmácia, Universidade de Lisboa, Lisbon, Portugal, Av. Prof. Gama Pinto, 1649-003 Lisboa, Portugal; marta-sereno@hotmail.com (M.S.); mvideira@ff.ulisboa.pt (M.V.); 2 Department of Galenic Pharmacy and Pharmaceutical Technology, Faculdade de Farmácia, Universidade de Lisboa, Lisbon, Portugal, Av. Prof. Gama Pinto, 1649-003 Lisboa, Portugal; 3 Institute of Biophysics, Biological Research Centre, Szeged, Hungary, Temesvári krt. 62, 6726 Szeged, Hungary; wilhelm.imola@brc.hu (I.W.); krizbai.istvan@brc.hu (I.A.K.); 4 Institute of Life Sciences, Vasile Goldiş Western University of Arad, Arad, Romania, Str. Liviu Rebreanu 86, 310414 Arad, Romania; 5 Department of Biochemistry and Human Biology, Faculdade de Farmácia, Universidade de Lisboa, Lisbon, Portugal, Av. Prof. Gama Pinto, 1649-003 Lisboa, Portugal

**Keywords:** biomarkers, blood–brain barrier, brain metastases, breast cancer, metastatic cascade, microRNAs

## Abstract

MicroRNAs (miRNAs) are small non-coding RNAs that mainly act by binding to target genes to regulate their expression. Due to the multitude of genes regulated by miRNAs they have been subject of extensive research in the past few years. This state-of-the-art review summarizes the current knowledge about miRNAs and illustrates their role as powerful regulators of physiological processes. Moreover, it highlights their aberrant expression in disease, including specific cancer types and the differential hosting-metastases preferences that influence several steps of tumorigenesis. Considering the incidence of breast cancer and that the metastatic disease is presently the major cause of death in women, emphasis is put in the role of miRNAs in breast cancer and in the regulation of the different steps of the metastatic cascade. Furthermore, we depict their involvement in the cascade of events underlying breast cancer brain metastasis formation and development. Collectively, this review shall contribute to a better understanding of the uniqueness of the biologic roles of miRNAs in these processes, to the awareness of miRNAs as new and reliable biomarkers and/or of therapeutic targets, which can change the landscape of a poor prognosis and low survival rates condition of advanced breast cancer patients.

## 1. Introduction

MicroRNAs (miRNAs or miR) are small non-coding ribonucleic acids (RNAs) that up until recently were fairly unknown, but that in the past few years have received great attention by the scientific community due to their unique properties and roles in many diseases [1,2,3,4]. The first miRNA discovered, *lin-4*, was found out in *Caenorhabditis elegans* (*C. elegans*) by Lee and colleagues, in 1993 [5]. It was identified as a small noncoding RNA (ncRNA) affecting development through regulation of the expression of the protein LIN-14. Seven years later, Reinhart et al. [6] reported another one in *C. elegans*, *let-7*, which negatively regulates the expression of the *LIN-41* gene through sequence-specific RNA–RNA interactions with the 3′-untranslated region (3′-UTR) of its mRNA. Subsequently, it was found out that they are abundant in both invertebrates and vertebrates, with estimates of 2300 true human mature miRNAs in 2019 [7], a number expected to increase in the years to come. It is worth noting that miRNAs are found in multiple genomes, with a high degree of species homology and sequences conserved across several species [8]. While little is known about their specific targets and biologic functions, there is evidence that miRNAs have crucial roles in the regulation of gene expression by controlling diverse cellular pathways [9] and changes in their expression have been associated with multiple human diseases [1,2]. The dysregulation of miRNAs has a prominent role in cancer and specific ones are either upregulated or downregulated in different types of the disease [10]. This has led to the creation of miRNA expression profiles that specifically correlate to each type of cancer even in early stages, allowing the detection and classification of poorly differentiated tumors and disclosing numerous possibilities in terms of diagnosis, prognosis and treatment [8]. In this review, we address the current knowledge and gaps in miRNA roles, focusing on their relevance in cancer development and spread and, particularly, in breast cancer (BC) and BC brain metastases (BCBM).

## 2. MiRNA in Health and Disease

### 2.1. MiRNAs Roles

It is predicted that up to 98% of the transcriptional output of the human genome represents RNA sequences that do not code for protein synthesis but fulfill several functions. MiRNAs are a subclass of single stranded small non coding (ncRNAs) with about 21–25 nucleotides that play important gene-regulatory roles by pairing to mRNAs of protein-coding genes to direct their posttranscriptional repression, hence having impact in crucial cellular functions [11]. Commonly, only a partial pairing between the miRNA and the target mRNA is enough to direct gene silencing. Therefore, a single miRNA can regulate several genes, while one single gene can be targeted by more than one miRNA [12], with estimates that about one third of the human genome is regulated by miRNAs [1]. Although until recently, miRNAs were believed to regulate gene expression exclusively by downregulating the target genes [13], recent studies have shown a dynamic alternation between downregulation or upregulation of target mRNAs, according to specific conditions, including cellular microenvironment, sequences and cofactors. Given the complexity of miRNA-mediated gene regulation, one single miRNA can act both in repression and stimulation of mRNAs. Accordingly, miR-145 has been described to upregulate myocardin during smooth muscle differentiation and proliferation [14], while the same miRNA downregulates Rho-associated protein kinase-1 (ROCK1) in cases of osteosarcoma [15]. Approximately half of the known miRNA sequences are found in close proximity to other miRNAs in the genome, forming clustered structures that have been hypothesized to regulate functionally related genes [16]. Moreover, some of the miRNAs present common structural and functional features and are categorized in different groups, named miRNA families [17].

MiRNAs play an evolutionarily conserved role in regulating numerous genes involved in development [18] and in crucial biologic processes [19]. In fact, animals that do not express miRNAs or have a deficient biogenesis fail to develop or survive [20,21]. It is now accepted that small variations in miRNA levels can have major cellular effects, and that their aberrant expression due to mutations, dysregulation or biogenesis dysfunction can lead to the blockade of physiological and biochemical pathways, resulting or being involved in pathologic outcomes [22]. Accordingly, ncRNAs are currently associated with a wide variety of human diseases, such as myocardial infarction, neurodegenerative diseases, as well as cancer [23,24], addressed below. In general, the loss or gain of function of miRNAs can result from chromosomal abnormalities, inherited mutations and single nucleotides polymorphisms in either the miRNAs or their targets, as well as from epigenetic silencing of primary miRNA transcription units or can be a consequence of defects in the biogenesis machinery [25,26]. MiRNAs processing can also be affected by other miRNAs, as described for miR-709 that can directly bind to its recognition element that is present on pri-miR-15a/16–1, interfering with its biogenesis and suppressing its maturation [27]. This leads to the possibility of a “microRNA hierarchy system” with a complex level of mutual interaction and regulation that may be involved in pathologic events.

Both in physiological and pathologic conditions, miRNA expression and their targets are, in many cases, tissue specific. In fact, different miRNAs are expressed in different tissues at different levels, with characteristic patterns that play an important role in tissue identity, differentiation and function. The same applies to pathologies, as specific miRNAs are aberrantly upregulated or downregulated in different diseases and can influence or even determine the pathologic phenotypes of cells [28]. Since this is a very recent subject, there are still no miRNA-based therapeutics approved. However, more than 20 clinical trials are in course. Among them, the tumor suppressor miR-34 encapsulated by lipid nanoparticles has reached phase I clinical trials for treating cancer and has shown to cause significant tumor reduction, while anti-miRs targeting miR-122 are now in phase II trials for treating hepatitis, and showed a great reduction in virus titers [29,30].

Although most miRNAs are found inside cells, they can also circulate in a cell-free form, commonly known as circulating or extracellular miRNAs (ECmiRNAs). These are found in body fluids, as blood, plasma, urine, cerebrospinal fluid, saliva and semen [31]. They are stable and can survive extreme conditions, including boiling, high or low pH and prolonged storage time, being also resistant to the abundant extracellular RNases [32,33]. These ECmiRNAs seem to be protected by encapsulation into membrane-vesicles [34], leading to the hypothesis of the existence of an intercellular crosstalk and inter-organ communication system mediated by extracellular vesicles, like exosomes, that can carry mRNAs and miRNAs, among other molecules [35]. Some miRNAs are preferentially loaded into exosomes, while others remain in the mother cells, indicating a selectiveness in the sorting process. The finding that exosomes and extracellular vesicles transport miRNAs to mediate intercellular and interorgan communication is remarkable and widely accepted constituting a landmark in the current disease pathophysiology knowledge.

Cell free miRNAs can also be shielded from extracellular environment by associating with high density lipoprotein or with argonaut-2 in a ribonucleo–protein complex. Although it remains unclear if and how these miRNAs facilitate cell–cell communication, they represent most circulating miRNAs [36]. Moreover, their unique properties fulfill all the characteristics of an ideal biomarker, as they are: specific and able to differentiate pathologies; sensitive because there is a quick and significant release upon the appearance of a pathology; predictive as they have long half-lives and should be proportional to the severity of the disease; robust and easily detectable by minimally invasive techniques; and, finally, translatable [37]. This, together with the fact that specific ECmiRNAs have already been associated with several diseases [38], raise the interest in further studies related to physiological or pathologic processes, as well as for their usefulness as reliable biomarkers to be used in liquid biopsies.

### 2.2. MiRNAs in Cancer

The recognition of miRNAs’ impact in cancer has driven the scientific community towards the elucidation of their role in the regulation of tumorigenesis. Around 50% of human miRNA genes are thought to be located nearby chromosomal breakpoints or in regions of instability, thus prone to gene deletion, amplification and mutations, all of which are considered cancer-associated regions or fragile sites of the genome [39]. The earliest evidence of miRNA involvement in human cancer came from Calin et al. [40] that demonstrated a frequent downregulation of miR-15 and miR-16 among B-cell chronic lymphocytic leukemia patients. Studies of the same authors further demonstrated that these miRNAs act as tumor suppressors by repressing Bcl-2, an anti-apoptotic protein overexpressed in malignant nondividing B cells [41,42]. In the last few years, miRNA profiling and deep sequencing showed that miRNA expression is dysregulated in cancer and that different tumors have specific miRNA signatures that can be used for tumor classification, diagnosis and prognosis, or even as therapeutic targets or agents [43,44]. MiRNA biogenesis machinery though not fully clarified, seems to include chromosomal abnormalities when copy numbers and gene locations of the miRNAs are altered (by amplification, deletion or translocation), transcriptional control changes when the transcription factors that control miRNAs’ expression like c-MYC or p53 are dysregulated, or epigenetic changes including DNA hypomethylation, hypermethylation and disruption of the histone modification patterns [45].

MiRNAs may play a role as oncogenes (oncomiRs), the most common type, by downregulating tumor suppressor genes and/or genes controlling cell differentiation or apoptosis [46]. In some cases, a miRNA can function as tumor suppressor or oncogene depending on the type of tumor or the stage of tumor progression. In fact, Costa-Pinheiro et al. [47] transfected two different prostate cancer cell lines with either miR- or anti-miR-375, and observed that forced expression of the miRNA in PC-3 cells attenuated the malignant phenotypes, whereas its forced inhibition in 22Rv1 cells resulted in the same effect, suggesting a dual role for miR-375 in carcinogenesis, acting as either an oncomiR or as a tumor suppressor.

### 2.3. MiRNAs in Metastatic Cancer

One of the hallmarks of tumorigenesis is the formation of metastases, an intricate process by which cells spread from a primary tumor to distant organs and tissues, forming viable secondary tumors. Recent studies showed that miRNAs have an active role in regulating metastases either presenting up- or downregulated expression, consequently decreasing or enhancing the expression of target genes [48]. Moreover, there are specific patterns of miRNAs expression in different cancers, as well as for the same type of cancer, depending on the colonized organ. Accordingly, a set of signatures both in primary and metastatic cancer cells, with several miRNAs universally expressed in different cancers, while others only expressed or aberrantly expressed in primary or metastatic sites, was established [49]. These observations indicate that miRNA expression is organ- and tumor type-dependent and point to their effectiveness as metastases biomarkers. In addition, circulating miRNA signatures in lung and breast cancer have been considered relevant cancer and metastases biomarkers not only for cancer diagnosis, but also for prognosis [50,51,52]. Moreover, Alhasan et al. [53] demonstrated that in human patients, very high risk prostate cancer has a unique circulating miRNA signature, different from the low risk forms of the disease.

Tumor cells often release higher numbers of microvesicles than other cells, and cancer patients present a higher quantity of serum exosomes than healthy individuals [54]. Accumulating evidence supports that horizontal transfer of exosomal factors, including miRNAs, can functionally influence stromal cells at distant sites, thereby facilitating tumor-stroma interactions and promoting the formation of a supporting metastatic niche in distant organs [55]. At this point, their aberrant expression in circulation can also be originated by the pre-metastatic niche cells [56] acting as selective players in a necessary cross-talk to promote a favorable environment for tumor seeding and growth in the host organ.

As cell free miRNAs can reach distant sites and regulate various cellular components of the tumor microenvironment, they may be important in understanding, detecting and targeting metastatic progression and, hence, valuable prognostic markers and targets for therapeutic intervention in specific types of tumorigenesis. Additionally, the presence of miRNAs that are associated with the process of metastases by regulating one or several of its steps may identify those patients that already have distant micrometastases that are too small to diagnose otherwise.

## 3. MiRNAs in Breast Cancer

### 3.1. Breast Cancer

BC is the most frequently diagnosed cancer in women worldwide and the leading cause of death from cancer in women, with almost 2.1 million new cases and 627,000 deaths in 2018, representing about 15% of all cancer deaths among women, according to the World Health Organization (https://gco.iarc.fr/). It is a malignant type of tumor that usually initiates in the epithelial cells of the mammary ducts [57]. Currently available treatments for early stage BC involve either a mastectomy or a lumpectomy, corresponding to a complete removal of the breast or only the tumor and some of the normal surrounding tissue, respectively, followed or not by radiation therapy and adjuvant treatments like chemotherapy or hormonal therapy [58]. Although these approaches are intended to prevent the recurrence of BC, there is the risk of relapse in distant organs, particularly when the malignancy is not early diagnosed, with up to 5% of patients presenting distal metastases at time of diagnosis and up to 15% within the first three years [59,60]. Additionally, growing evidence suggests that depending on the histopathologic and biologic features, BC exhibits distinct behaviors that result in different responses to treatment [61]. Thus, besides the classical classification that stratifies tumors into four stages according to tumor size, regional nodal involvement and distant metastases, other types of classification that consider non-anatomic characteristics like biomarkers have been widely used [57]. Nowadays, the most used classification, subdivides breast tumors according to the expression of human epidermal growth factor receptor 2 (HER2), progesterone receptor (PR) and estrogen receptor (ER), whereas absence of all the receptors expression corresponds to the triple negative type [57]. This type accounts for approximately 15–20% of all BCs and is usually associated with the worst prognosis, due to its high rate of relapse, tendency to form metastases in visceral organs and current lack of targeted therapies [62,63].

Since miRNAs aberrant expression in BC was first described in 2005 [41], different stages of BC were correlated with distinct miRNA profiles, suggesting that they are directly involved in tumor progression and metastases [64]. Moreover, miRNA profiling studies have led to the identification of miRNAs that are deregulated during the several stages of BC metastases, reinforcing the potential of miRNAs as diagnosis and prognosis biomarkers [65]. Importantly, it has been demonstrated that restoring the expression of certain miRNAs that are usually downregulated in BC models can suppress metastases in vivo [66]. However, it remains to be established the relationship between deregulated miRNAs in metastatic BC and the primary tumor subtype for most of the miRNAs. In fact, apart from the association between downregulation of miR-520c and formation of lymph node metastasis in ER- patients [67], the relationship between several tumor suppressor or metastases promoter miRNAs and the primary BC type are usually not addressed [68,69,70], representing a lack that should be fulfilled.

### 3.2. MiRNAs throughout the Metastatic Cascade in Breast Cancer

**Figure 1:**
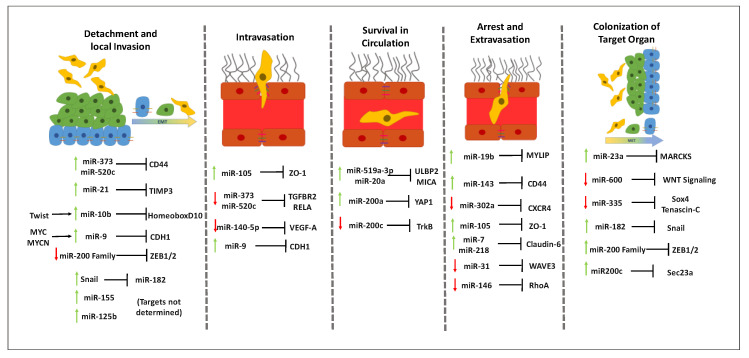
Simplified representation of the metastatic cascade of breast cancer and of the microRNAs (miRNAs; miR) involved in each of the steps of the process. This multistep process comprises: (1) detachment and local infiltration of malignant cells into the surrounding tissue, which involves phenotypic changes with loss of epithelial features such as the adhesion of neighboring cells and gain of mesenchymal features that endow cells with migratory properties, a process known as epithelial-mesenchymal transition; (2) intravasion, corresponding to the transendothelial migration of breast cancer cells to reach the circulation; (3) circulation and survival in the blood stream; (4) arrest and extravasation into the target organ, involving the transendothelial migration across the endothelium; and (5) proliferation and colonization of competent organs, involving the gain of the epithelial characteristics and loss of the mesenchymal ones, a process known as mesenchymal-epithelial transition. The miRNAs that have been associated with each of the steps of the metastatic cascade are represented. Those working as oncogenes are usually upregulated (green arrows), while the ones that work as tumor suppressors are usually downregulated (red arrows). Known downstream targets and upstream regulators of miRNAs are also indicated.

In order to spread from the breast to different organs, BC cells (BCCs) need to undergo a series of steps, commonly named as the metastatic cascade [57]. This multistep process comprises: (1) local infiltration of malignant cells into the surrounding tissue; (2) intravasion, which is the transendothelial migration (TEM) of BCCs into vessels to reach the circulation; (3) circulation and survival in the blood stream; (4) arrest and extravasation to the target organ; and (5) proliferation and colonization of competent organs. In this section, we dissect the current knowledge about the several steps of the metastatic process and the evidences pointing to miRNAs involvement, which are summarized in Figure 1.

#### 3.2.1. Influence of MiRNAs in Detachment and Local Invasion of Malignant Cells

Under normal conditions, the architecture of the mammary epithelium is ensured by cell–cell and cell-basement membrane (BM) interactions. Cell–cell interactions are established through intercellular junctions, including tight junctions (TJs) and adherens junctions (AJs) [57]. During transformation of normal epithelial cells into BCCs cell–cell and cell-BM adhesion are disrupted allowing tumor cells detachment and invasion of the surrounding tissue. These processes simultaneously require cell plasticity, increased motility and ability to remodel the extracellular matrix (ECM) [71]. This is partially achieved by the epithelial-mesenchymal transition (EMT), a process characterized by a phenotypical change from cuboidal to an elongated spindle shape, with loss of cell–cell and cell-BM adhesion and gain of migratory capacity [57]. These changes include the loss of epithelial markers, as E-cadherin, a transmembrane glycoprotein that forms the core of the AJs that maintain cell–cell adhesion, as well as increased expression of mesenchymal markers, including N-cadherin, vimentin, smooth-muscle actin and cadherin-11 [72]. The switch between E-cadherin and N-cadherin, which has been widely used to monitor the progress of EMT, increases BCCs motility and invasiveness [73] that in turn is facilitated by changes in the ECM surrounding tumor cells [74].

Numerous miRNAs have been described to regulate EMT [75] and to be involved in BCCs detachment and local invasion. Particularly in BC, the miR-200 family members (miR-141, miR-429, miR-200a, miR-200b and miR-200c) have shown to be powerful regulators of EMT, by being highly expressed in epithelial cells and downregulated in cells with mesenchymal phenotype [76]. This family promotes the epithelial state by downregulating epithelial gene transcriptional repressors ZEB1/ZEB2 [77]. ZEB1/ZEB2 are known to induce EMT by strongly suppressing the expression of E-cadherin, while ZEB2 directly activates vimentin [78]. Several other miRNAs, including, miR-155, miR-10b, miR-21 and miR-125b have been shown to act as promoters or repressors of EMT in BC, through diverse mechanisms and signaling pathways. In fact, the remodeling of the ECM by metalloproteinase (MMP)3 was shown to be promoted by miR-21 by inhibiting the tissue inhibitor of MMP3 (TIMP)3, which is secreted in large quantities by BCCs and degrades the ECM, facilitating local invasion and migration of malignant cells [79]. Accordingly, high expression of miR-21 has been considered a risk factor and an indicator of bad prognosis in BC patients [80]. miR-10b has also been associated with BC invasion and metastases initiation. The high expression of miR-10b in metastatic BC is induced by the transcription factor Twist, which binds directly to the putative promoter of mir-10b and leads to the inhibition of translation of the messenger RNA encoding homeobox D10, resulting in increased expression of a well-characterized pro-metastatic gene, *RHOC.* Thus, Twist-mediated miR-10b upregulation induces local invasion and migration of BCCs [68]. MiR-373 and miR-520c promote detachment of BCCs by mediating loss of cell-ECM interactions by downregulating CD44 [81], a cell surface receptor for hyaluronan, one of the major components of the ECM [82]. Another miRNA that is upregulated in BCCs is miR-9, which promotes loss of cell–cell interactions by targeting *CDH1*, the gene that encodes for the epithelial cell adhesion molecule E-cadherin. The oncoproteins MYC and MYCN are responsible for the upstream regulation of miR-9 by acting on the mir-9–3 locus, causing activation of miR-9 expression in tumor cells [83]. Thus, by downregulating E-cadherin, miR-9 can also be involved in the regulation of EMT. Collectively, by affecting EMT and BCC invasion, miRNAs can be determinant to the patient’s prognosis and can constitute valuable therapeutic targets [84].

#### 3.2.2. Involvement of MiRNAs in Intravasation

Once malignant cells undergo EMT, detach from the primary tumor and invade the surrounding tissue, to reach distant organs they need to enter the circulatory or lymphatic system in a process named intravasation [57]. The hematogenous route is the most common one, mainly because of the higher accessibility of blood vessels, since tumor angiogenesis creates a network of microvasculature that is accessible to malignant cells [85]. Similar to epithelial cells, the endothelial cells that line the blood vessels are also connected by cell–cell junctions, namely by TJs [86]. Thus, for intravasation to occur, BCCs must induce molecular and cellular changes to overcome these blockades and cross the endothelial barrier. Although some studies showed that TEM can occur by a transcellular route, in which the BCCs transmigrate through individual cells, the preferred route for TEM seems to be the paracellular route, in which BCCs transmigrate through interendothelial junctions, by disrupting their integrity [87,88]. In BC, miR-105 has a relevant role in this disruption, as exosome-mediated transfer of cancer-secreted miR-105 efficiently destroys TJs and the integrity of vascular endothelial barrier, thus promoting metastases [89]. MiR-105 directly targets zonula occludens-1, one of the TJ proteins [86]. Accordingly, overexpression of miR-105 in non-metastatic BC cell lines, induces formation of metastases and increased vascular permeability in distant organs [89]. Furthermore, the expression of the miR-520c/miR-373 family negatively correlates with lymph node metastases of BC [67]. This family of miRNAs, particularly miR-520c and miR-373 inhibit in vivo intravasation of BC by directly suppressing *TGFBR2* and *RELA*, leading to a downregulation of transforming growth factor β (TGF-β) and nuclear factor (NF)-kB, respectively. TGF-β reduction leads to a decrease in angiopoietin-like 4, a protein known to disrupt vascular integrity through targeting vascular endothelial cadherin and claudin-5, well known components of AJs and TJs, respectively [67,81].

The several members of the vascular endothelial growth factor (VEGF) family (VEGF-A, -B, -C, -D and -E and placental growth factor) and its receptors promote metastases and intravasation, through augmenting tumor microvasculature availability due to the powerful angiogenic activity [85,90]. Moreover, VEGF-A is released by tumor associated macrophages to disrupt cell–cell interactions, mainly TJs, thus increasing vascular permeability [91,92]. MiR-140-5p was reported to suppress VEGF-A and inhibit angiogenesis, as well as to downregulate MMP9 expression and reduce BCCs invasion; moreover, it is decreased in human BC samples and BC metastasis comparing with the corresponding adjacent normal tissues and cancer without metastasis. Thus, this miRNA seems to function as a tumor suppressor by inhibiting VEGF-A-mediated BCCs invasion and metastasis development [93]. In turn, miR-9, a well-known oncomiR, was shown to be highly expressed in cell lines. This overexpression is induced by MYCN transcription factor and leads to the direct inhibition of E-cadherin. MiR-9-mediated E-cadherin downregulation results in the translocation of β-catenin to the nucleus, where it promotes the transcription of the gene encoding for VEGF-A thus leading to increased tumor angiogenesis [83]. Such results suggest a dual role of miR-9 by inducing EMT and facilitating intravasation. Other miRNAs have been suggested to modulate endothelial cells activities and be involved in BC angiogenesis, including miR-216a, miR-330, miR-608, miR-10b, miR-196b, miR-27a and miR-19 [94].

#### 3.2.3. Role of miRNAs in Circulating Tumor Cells Survival in Circulation

BC metastatic cells that succeed in intravasation, either via the blood or lymphatic circulation, become circulating tumor cells (CTCs). Throughout their path, CTCs can encounter many obstacles, including shear forces of the circulation, collision with host cells and attack of the immune system. All these factors influence CTCs survival and limit their ability to establish metastases in distant sites. In fact, millions of metastatic cells are shed by the tumor to the bloodstream, but only a small percentage reaches the target and very few clinically relevant metastases are formed, compared to the number of cells released by the tumor. Thus, it is expected that a selection of the most resistant and aggressive tumor cells occurs [95]. The first and most relevant immune response against the CTCs is played by natural killers (NKs), despite the fact that tumor development is accompanied by a dysfunction and reduction of cytotoxicity by NKs, induced by BC CTCs [96,97]. Breunig et al. [98] demonstrated that miR-519a-3p can efficiently impair tumor cell killing by NKs and confer BCCs resistance by targeting TRAIL, FasL and granzyme B. The authors further showed that this is achieved via the downregulation of UL16-binding protein 2 (ULBP2) and major histocompatibility complex class I chain-related protein A (MICA) in the surface of tumor cells. ULBP2 and MICA are natural killer group 2 member D (NKG2D) ligands and are crucial for recognition of BCCs by NK cells. Accordingly, high levels of miR-519a-3p are associated with poor survival of BC patients, possibly by increasing the survival of CTCs in circulation [99]. MiRNAs belonging to the miR-17-92 cluster, especially miR-20a, have also shown to decrease the expression of MICA and ULBP2 by targeting the MICA 3′-UTR and by inhibiting the MAPK/ERK signaling pathway, respectively. Likewise, the silencing of NKG2DL-targeting miRNAs, including miR-20a in BCCs, increased NK cell-mediated cytotoxicity in vitro and inhibited immune escape in vivo [100]. Another important feature that malignant cells must acquire to survive within blood and lymphatic circulation is the resistance to anoikis, a particular form of cell death occurring by loss of interactions between epithelial cells and ECM [101]. Interestingly, an increased sensitivity of BCCs to anoikis is determined by one of the members of the miR-200 family, miR200c, which is usually downregulated in BCCs with a mesenchymal phenotype. Neurotrophic tyrosine receptor kinase type 2 was suggested as the direct target that mediates this effect [102]. Contrarily, Yu et al. [103] showed that a member of the same family, miR-200a, promotes resistance to anoikis and, consequently, lymph node and distant metastases in BCCs. The proposed mechanism is through direct targeting of the 3′-UTR of the gene that encodes for Yes-associated protein 1 (YAP1) by miR-200a. YAP1 knockdown in BC cell lines suppressed anoikis and increased migration and invasiveness, suggesting its role as a tumor suppressor, which is in line with the decreased expression of YAP1 in BC patients [104]. Altogether these findings suggest that the family of miR-200, which is highly associated with EMT in BC and the promotion of distant metastases [76,105], can also have a relevant role in the survival in circulation of CTCs through the regulation BCCs resistance to anoikis.

#### 3.2.4. Effect of MiRNAs in the Arrest and Extravasation of Malignant Cells

The cells that survive the hostile intravascular environment need to cross the vascular endothelium to extravasate into the surrounding tissue. The extravasation process involves adhesion to the endothelium, modulation of the endothelial barrier and, finally, TEM to reach the surrounding tissues. Once the cells reach the tissues, they can start proliferating again to form new solid tumors. Extravasation of BCCs usually takes place in the microvasculature near the target site, in a process similar to the mechanism proposed for leucocyte extravasation during inflammatory response, from the luminal to the abluminal side of the endothelium [57]. Similar to intravasation, the preferred route for cancer cells to transmigrate through the endothelial barrier seems to be the paracellular route, which is the better studied [106,107]. The adhesion and arrest of BCCs at the endothelium is a crucial step in the extravasation that is mediated by the interaction of numerous ligands and receptors, including selectins, cadherins, integrins, the cells surface adhesion receptor CD44 and immunoglobulin superfamily receptors, but also chemokine receptors like C-X-C chemokine receptor (CXCR) type 4 (CXCR4) or type 7 (CXCR7) [108,109].

MiR-19b, a member of the miR-17–92 cluster, is a key oncomiR in BC by regulating the PI3 K/Akt pathway and leading to the downregulation of several tumor suppressor genes, including phosphatase and tensin homolog (PTEN) [110]. Recently, miR-19b was found to be upregulated during BC metastases. This event was directly related with the downregulation of miR19b’s target, myosin regulatory light chain interacting protein, an upstream event for the downregulation of E-cadherin and upregulation of intercellular adhesion molecule-1 and integrin β1, all molecules involved in the adhesion process. These observations suggest a potential role for miR-19b in adhesion of BCCs to the endothelium during extravasation [111]. As previously mentioned, CD44 expression strongly correlates with cancer cell adhesion to endothelial cells and with cancer metastases. Specific glycosylated forms of CD44 present in BCCs bind to the vascular adhesion molecule E-selectin present in endothelial cells, promoting adhesion and TEM of ER-/CD44 + BCCs [112]. Recently, miR-143 was shown to inhibit tumor progression of BC metastatic cells, both in vivo and in vitro, by directly targeting CD44. Accordingly, miR-143 was subsequently shown to work as a tumor suppressor, through its interaction with CD44, despite the role of CD44 in the maintenance of cancer stem cells properties [69]. Regarding miR-302a, it was downregulated in highly metastatic BCCs both in vitro and in vivo and its upregulation inhibited BC metastases. Although no concrete mechanism was proposed, this tumor suppressor activity of miR-302a was correlated with the downregulation of CXCR4 [70]. CXCR4 is known to be involved in extravasation of metastatic BCCs to their target organs through the binding of its ligand C-X-C motif chemokine 12 (CXCL12), also known as stromal cell-derived factor 1. This molecule is produced by endothelial cells of the microvasculature and stroma of certain target organs, including lung, liver and brain, thus having a chemoattracting role and promoting the adhesion of BCCs to the endothelium. The CXCR4/CXCL12 axis has also been implicated in vascular permeability, endothelial cell patterning and morphology and TEM of BCCs [113]. MiR-105 not only destroys endothelial barriers in primary sites, but when secreted by BCCs can also promote extravasation by downregulating zonula occludens-1 and, consequently, disrupting TJs in secondary sites. The disruption of TJs facilitates the TEM of BCCs, through paracellular route and consequently extravasation. In fact, the overexpression of miR-105 in non-metastatic BCCs augments vascular permeability and distant metastases, while the inhibition of the same miRNA in highly metastatic tumors lessens these effects [89]. Both miR-7 and miR-218 produced by BCCs were shown to inhibit the expression of another TJ protein, claudin-6, thus suggesting a possible role for miR-7 and miR-218 in promoting the modulation of endothelial barriers and the paracellular route of TEM in BC [114,115]. Still, for CTCs to transmigrate they need to display a certain degree of deformability. In a triple negative model of BC this was shown to be directly correlated with the overexpression of transient receptor potential vanilloid subtype 4, a calcium permeable channel, capable of inducing reorganization of the actin cytoskeleton, and, consequently, reducing cell rigidity and promoting motility and extravasation of metastatic BCCs [116]. Some miRNAs can be involved in the remodeling of the actin cytoskeleton during extravasation. MiR-31was shown to inhibit the migration and invasion of triple negative BCCs by suppression of the expression of special AT-rich sequence-binding protein 2 (SATB2), a protein known to regulate the expression of genes involved in actin dynamics [117]. This ability was later on related with miR-31-mediated inhibition of WAVE3, an actin cytoskeleton remodeling protein that is highly expressed in advanced stages of BC and influences cancer cells motility, invasion and metastases [118]. WAVE3 also has an established role in earlier stages of the metastatic cascade due to its regulation of EMT, where miR-200c is responsible for its regulation [119]. Rac1, Cdc42 and RhoA are some of the members of the RhoGTPases family that is involved in signaling pathways related to actin cytoskeleton organization and cell motility. They are upregulated in tumors, contributing to cancer cell migration, invasion and metastasis [120]. The expression of RhoA was inversely correlated with that of miR-146 in BC cell lines and it was suggested that this miRNA functions as a tumor suppressor in BCCs, which was corroborated by the fact that its downregulation increased the migration of BCCs via upregulation of RhoA [121].

#### 3.2.5. Contribution of MiRNAs to the Colonization of the Target Organ

The last step of the metastatic cascade is the colonization and establishment of macroscopic secondary tumors at distant organs [57]. CTCs that can successfully cross endothelial barriers and infiltrate the target organs are called disseminating tumor cells (DTCs). Although these cells were successful in entering a secondary organ, they still face some obstacles to adapt to a new microenvironment. Indeed, organ colonization is a key rate-limiting step of the metastatic process [122] and there is a “lag time” between tumor dissemination and metastatic dissemination commonly referred to as metastatic dormancy [123]. This period corresponds to DTCs adaptation to the new environment, during which cells reside in the target organ as single cells or as micrometastases, in which either proliferation and apoptosis occur at similar rates or single-infiltrated cells are blocked in the G0 phase of cell division cycle to stay in a state of proliferative quiescence [123]. It has been proposed that for remaining in this state, DTCs are in a stemness state and have similar characteristics to adult stem cells that reside within the organs [124]. These cells regain the ability to proliferate upon a certain stimulus. In effect, signals from the microenvironment influence the behavior of DTCs and determine whether they stay dormant or if proliferation pathways are activated for the cells to form macrometastases [125].

MiRNAs seem to have an important role in the switch between dormant and activated BCCs. Exosomes released by bone marrow stem cells induce dormant phenotypes of BC metastatic cells by releasing miR-23a, which suppresses the target gene *MARCKS* that encodes for a protein responsible for the promotion of cell cycling and motility. Thus, exosomal transfer of miRNAs can promote BCC dormancy in a metastatic niche [126]. On the other hand, miR-138 and miR-346 overexpression in BC DTCs regulates their metastatic reactivation, indicating that these miRNAs can promote exit from dormancy in the lung [127], remaining to clarify the mechanism underlying this regulation. MiR-600 is capable of regulating BC stem cells fate, as silencing of this miRNA results in the acquisition of a BC stem cell phenotype, characterized by the ability of self-renewal of BC cells, as well as their arrest in an undifferentiated state. In turn, overexpression of miR-600 reduces their proliferation and self-renewal by blocking Wnt signaling, which indicates a possible role for miR-600 in the dormancy of DTCs [128]. Aberrant Wnt signaling has been observed in many types of cancer and has been implicated in triple negative BC tumorigenesis and metastasis as triple negative BC patients that display dysregulated Wnt signaling are more prone to develop lung and brain metastases [129].

Once DTCs have adapted to the microenvironment and established an accommodating metastatic niche, they start proliferating again to colonize and invade the secondary sites. In this step of the metastatic cascade, the production of MMPs and cathepsins is essential to promote the rearrangement of the ECM and drive cell invasion and migration through the stroma [130]. To support metastatic development, several growth and survival pathways are activated, including PI3 K/Akt, MAPK, Notch and Wnt signaling pathways [131]. Finally, to complete colonization of the target organ, BCCs must reacquire an epithelial phenotype, so they undergo the opposite process of EMT, known as mesenchymal-epithelial transition (MET) [132], which is far less investigated than EMT. During this process, BCCs regain the expression of epithelial proteins like E-cadherin and downregulate the expression of mesenchymal ones like N-cadherin and vimentin, thus switching from a non-differentiated state, characterized by proliferative quiescence, to a differentiated state, characterized by active proliferation [124,133].

As in all stages of the metastatic cascade, miRNAs can have an active role in promoting or inhibiting the colonization of distant organs. MiR-335 is a well-known tumor suppressor that was found absent in primary breast tumors from patients who relapsed and was associated with a poor metastasis-free survival, in line with its role in inhibiting migration, invasion and metastatic colonization by regulating several metastasis-implicated genes, such as *SOX4* and *TNC* [134]. Accordingly, reduced expression of miR-335 in BCC was associated with overexpression of the ECM component tenascin C that supports the survival and outgrowth of metastasis by activating pro-tumorigenic pathways like Notch and Wnt signaling [135]. Furthermore, the downregulation of miR-335 in metastatic BC is epigenetically regulated, particularly by the hypermethylation of a specific CpG island upstream of the transcriptional site of miR-335 promoter [136]. MiR-182 is downregulated in earlier stages of BC metastases due to its role as a suppressor of EMT as it is directly repressed by snail to promote EMT. In contrast, in advanced stages of metastasis development it is upregulated and inhibits snail to reestablish epithelial identity of BCCs, indicating a dual role of this miRNA during BC metastasis formation and a dynamic reciprocal suppression between miR-182 and snail [137]. As previously mentioned, the miR-200 family members have relevant roles along several steps of the metastatic cascade in BC. In fact, miR-200c is overexpressed and promotes BC metastatic colonization by directly targeting Sec23a, which mediates secretion of metastases suppressive proteins, including insulin-like growth factor-binding protein and Tinagl1. Moreover, miR-200s family members promote BC metastatic colonization by inducing MET, through the targeting of ZEB-1 and ZEB-2 that are repressors for the epithelial marker E-cadherin [76,138]. Altogether, these findings indicate that miRNAs are involved in the development of well-established metastases. However, when the secondary tumor reaches a certain dimension, nutrients and oxygen become scarce. To overcome this problem, new blood vessels are originated through the sprouting of preexisting vessels surrounding the tumor and miRNAs can also be involved in the regulation of angiogenesis in cancer [139].

## 4. MiRNAs in Breast Cancer Brain Metastases

### 4.1. Breast Cancer Brain Metastases

With the improvements in primary BC treatment, the prognosis of patients is considerably worsened by the appearance of metastasis that are responsible for 90% of human cancer deaths [140]. BC is the second most frequent contributor of brain metastases, following lung cancer, but considering the higher incidence of BC [141], brain metastases from BC assume a great relevance. According to estimates, 10–16% of patients with stage IV BC will develop brain metastases, plus an additional 10% of asymptomatic patients as revealed by autopsy studies [142,143]. The most common symptoms of brain metastases comprise constant headache, seizures, motor weakness, ataxia, altered mental status and dysphasia, which drastically impair the quality of life of both patients and their families [144,145]. It is also worth noticing that there are several risk factors that increase a BC patient’s probability to develop brain metastases, including younger age, ductal histology, increased tumor size, nodal metastases, lung and liver metastases, higher tumor grade, overexpression of epidermal growth factor receptor (EGFR), mutations in the *BRCA1* gene and the overexpression of Ki-67. Among the several types of BC, HER2-positive and triple negative tumors are the ones presenting the highest predisposition to brain metastases and are thus considered the most aggressive types and the ones with the lowest survival rates [146,147], as aforementioned. Current treatment for brain metastases includes open surgical resection, gammaknife or cyberknife stereotactic radiosurgery, focused external beam radiotherapy, whole-brain radiotherapy, traditional chemotherapy and newer targeted biologic agents personalized for tumor type, although none of them has shown to be completely effective and only help to diminish symptoms and prolong the patients’ life expectancy [148,149]. Therefore, brain metastases have been associated with the worst prognosis, with medium survival after diagnosis of brain metastases ranging from 2 to 16 months, depending on the study and on the involvement of the central nervous system, the extent of the extra-cranial metastatic disease and the treatment applied [150]. The best outcomes are achieved in patients with good performance status and single brain lesion and are presently increasing with the improved HER2-targeting therapies [151].

Despite its clinical importance, the molecular mechanism of brain metastases is still poorly understood. The complex process of formation of brain metastases comprises all the steps of the metastatic cascade plus the colonization and growth in the brain parenchyma. This process was reported to take 3.72 years for HER2-positive and 2.07 years for triple negative BC types [152]. Thus, BCBM are considered a late event because cancer cells must develop the ability to penetrate through the blood–brain barrier (BBB) and colonize the brain [146]. This contributes to the poor efficiency of treatments targeting brain metastases because most patients have already received several rounds of chemotherapy before its detection, which allows cancer cells to accumulate enough mutations to become resistant to new approaches [153,154].

Once BCCs enter the brain, they encounter an ideal microenvironment for metastatic growth, since the BBB provides protection against immune surveillance, chemotherapeutic agents and other harmful substances [155]. The anatomic basis of the BBB consists of brain microvascular endothelial cells that, not only form elaborate TJs, but also use active efflux transport mechanisms that restrict the entrance of molecules into the brain [156], thus blocking drugs from reaching metastatic sites. Regarding the route of BCCs migration across the BBB endothelium, the transcellular pathway has been the mostly described, with evidences of the redistribution of TJs proteins and increased BBB permeability [57,157]. However, recent evidences based on transmission electron microscopy analysis further showed the involvement of the transcellular path [158]. In a genome wide comparative study, several proteins have been proposed as mediators of the TEM of BCCs through the BBB, including the EGFR ligand HBEGF, cyclooxygenase-2 and α-2,6-sialyltransferase 5, with the latter specifically acting as a specific mediator of BCCs infiltration through the BBB [159]. Moreover, other proteins have a relevant role in the interactions between BCCs with the brain endothelium, including TJ proteins, selectins, integrins, cadherins, Rho GTPases and VEGF [160].

Although the brain microenvironment can be very hostile and kill most of the metastatic cells in a first response [161], the few cells that survive can benefit from the shielding and the cells present in the brain microenvironment can switch to a supporting role. Indeed, there is a crosstalk between malignant cells and brain cells, rendering the brain a sanctuary against anti-tumor strategies [162]. The brain tumor microenvironment consists of a complex network of interactions between astrocytes, endothelial cells and microglia [163]. Astrocytes are among the first brain cells to encounter extravasated malignant cells and the main determinants of their fate. Indeed, active astrocytes are found in close proximity to BCCs even before the extravasation process is finished [164]. Astrocytes contribute to brain metastases from BC by producing factors, including ERK1/2 and TIMP2 that activate MAPK signaling pathways in malignant cells. The overactivation of MAPK leads to the increased expression of MMP2 by tumor cells [165]. In turn, BCCs release interleukin-1β (IL-1β) which activates the surrounding astrocytes. The activation by IL-1β also augments the production of JAG1, which stimulates Notch signaling by BC stem cells, promoting their self-renewal [166]. In contrast, plasmin from the reactive brain stroma can be toxic for malignant cells by converting membrane-bound astrocytic FasL into a paracrine death signal for breast and lung cancer cells. The metastatic cells fight the production of plasmin by expressing high levels of anti-PA serpins, including neuroserpin and serpin B2 [167]. Altogether, these findings reinforce the idea of a crosstalk between brain cells, namely astrocytes, and malignant cells to support metastatic growth. Microglia, along with astrocytes, are the glial cell types most associated with brain metastases [168]. Microglia are the resident immune cells of the central nervous system that are non-proliferative in normal adult brain but can be rapidly activated in pathological conditions like neoplastic tumors [169]. In contact with tumor cells, microglia cells secrete a multitude of factors that can modulate the tumor microenvironment and enhance the colonization of tumor cells [170]. Among the secretome of microglia, there are mRNAs and miRNAs that can regulate the expression of genes in other cells, including tumor cells [171]. The exchange of released factors between microglia and tumor cells activates multiple key signaling pathways, including the Wnt signaling that needs to be active during microglia-induced invasion [172]. It was also reported that metastatic BCCs have a high expression of neurotrophin-3, which has a dual function of regulating the growth of metastasizing BCCs and of reducing the activation of immune response in the brain, by decreasing the number of fully activated cytotoxic microglia [173]. Moreover, there is a crosstalk between microglia and astrocytes that leads to microglia activation. This is mediated by the binding of STAT3+ reactive astrocytes associated with brain metastases that present an increased expression of macrophage migration inhibitory factor (MIF), which binds to CD74+ microglia that contributes to the establishment of an immunosuppressive microenvironment. Such activated microglia present upregulation of midkine, a downstream target of NF-kB signaling pathway that promotes the development of brain metastases [174]. The acquisition of neurons-like properties by BCCs can also support brain metastases development. In fact, BCCs acquire features similar to neurons like the overexpression of many variables related to γ-aminobutyric acid (GABA) (e.g., GABA_A_ receptor, GABA transporter and GABA transaminase). Such acquisition of a GABAergic phenotype renders the malignant cells able to take up and catabolize GABA as a biosynthetic source, indicating that they co-opt GABA as an oncometabolite to obtain energy for cell proliferation [175]. Moreover, BCCs metastasizing the brain express high levels of n-methyl-d-aspartate receptor and obtain its major agonist, l-glutamate, from glutamatergic synapses where high levels of the neurotransmitter are released from excitatory pre-synaptic neurons. To access glutamate, the tripartite synapses formed by astrocytes and pre- and post-synaptic neurons are subverted by metastasizing cells that take the place of astrocytes forming pseudo-tripartite synapses [176]. There is much less information about the role of pericytes in brain metastases from BC, although it has been proposed that during BCBM progression, there are different subpopulations of pericytes that can regulate the permeability of the BBB. The subpopulations are distinguished by the presence of certain proteins like desmin and CD13 [177].

### 4.2. MiRNAs Involved in Metastasizing Breast Cancer to the Brain

**Figure 2:**
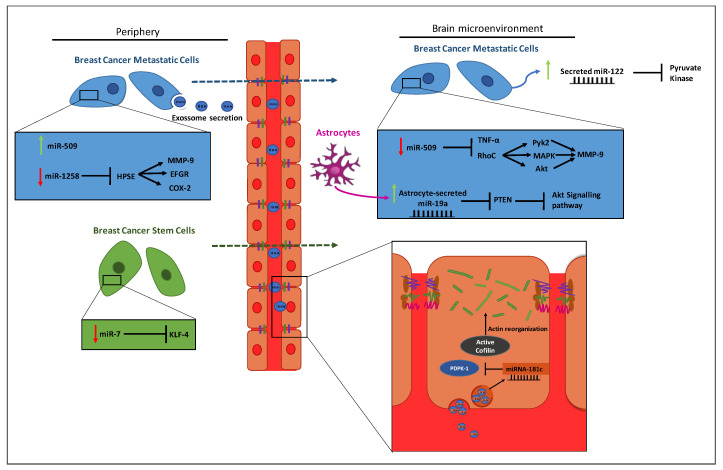
MicroRNAs (miRNAs; miR) currently associated with breast cancer brain metastases. Changes in the expression of miRNAs that have been correlated with the metastatic process, as well as their putative targets, are shown in different types of cells, including breast cancer cells, breast cancer stem cells, endothelial cells and cells in the brain microenvironment, namely astrocytes, are shown. While some miRNAs act intracellularly, other are released, either directly to the extracellular environment or encapsulated by exosomes that can then be transported to further places. In the insets, the intracellular mechanisms are schematized. It is known that miR-122 acts in the pre-metastatic niche, but the specific types of cells were not described yet.

Considering the difficulties in the treatment of brain metastases, an early diagnosis increases the chances of survival. However, the resolution of current MRI techniques and the contrast agents currently used do not allow an efficient detection of small tumors or micrometastases, which could be targeted by first-line treatment more efficiently than macrometastases [178]. As previously mentioned, miRNAs may serve as effective new biomarkers in predicting cancer progression, given the fact that metastatic cancer cells express specific miRNAs. Due to the high incidence, poor prognosis and devastating consequences of BCBM, the use of miRNAs as biomarkers is currently being studied and is of high clinical interest. Several miRNAs were already associated with different steps of the metastatic process [179], and some of the putative targets were identified (Table 1). Since this is a recent subject, few studies have been performed and the downstream targets of the microRNAs and the mechanisms by which they act are still uncertain and up to discussion. In this review, we will focus on the miRNAs for which there is information available and whose main features are depicted in Figure 2.

Metastatic cancer stem-like cells (CSCs), which are associated with tumor growth and metastasis initiation due to their properties like cell growth, cell cycle and self-renewal and invasiveness, are highly regulated by miRNAs [190]. Accordingly, it has been demonstrated that miR-7 is significantly downregulated in CSCs from a breast cancer cell line that metastasizes to brain, as compared with the parental nonmetastasizing line, suggesting a specific function of this miRNA in metastatic cells; moreover, the expression of miR-7 was inversely correlated with that of its downstream target, KLF4, one of the genes responsible for maintaining stem cells properties [180]. The promotion of BCBM by miR-7 may further be related to downstream targets of KLF-4, like TGF-β and Notch that are known to be involved in stem cell self-renewal and tumor progression or with microenvironmental factors in the brain [180,190].

Zhang et al. [191] demonstrated that miR-1258 is downregulated in BCBM and that its ectopic expression results in the inhibition of cell invasion and onset of brain metastases, indicating that this miRNA is a suppressor of brain metastatic BC. It was also shown that its tumor suppressor action occurs by downregulation of heparanase (HPSE), a mammalian endoglycosidase with tumorigenic, angiogenic and pro-metastatic activity that is highly expressed in cancer cells with high propensity to colonize the brain [189,191]. In effect, this is not surprising considering that HPSE plays a critical role in BC progression, particularly in cell proliferation, angiogenesis, invasion and metastasis formation due to the degradation of heparan sulfate that allows the release of growth factors from the cell surface and ECM [192]. HPSE-mediated promotion of BCBM can be related with its downstream targets MMP9, cyclooxygenase-2 and EFGR, given the fact that inhibition of HPSE resulted in their decreased expression levels in a BCBM model. It is worth noticing that these proteins were recently related with brain metastases due to their important roles in the disruption of the BBB [193,194,195]. Although a treatment with miR-1258 reduces BCBM by inhibiting HPSE, there are cross-talk mechanisms between tumor and normal cells of the brain microenvironment, like the production of HPSE by astrocytes [196], that cannot be inhibited by miR-1258 since it only targets the intracellular production of HPSE by BCCs.

MiR-509 has been described to be highly expressed in primary breast tumors whereas its expression is significantly decreased in brain metastatic lesions originated from the same tumors. Moreover, the levels of this miRNA are decreased in primary breast tumors of patients with brain metastases when compared with BC patients without this kind of metastatic lesions. The downregulation of miR-509 directly relates with the upregulation of both RhoC and TNF-α, indicating that the expression of these proteins is regulated by such miRNA [186]. RhoC is known to enhance the migration and invasive ability of BC stem cells by activating several pathways, thus, impacting its metastatic potential and frequency [197,198]. Xing et al. [186] suggested that miR-509 suppresses brain metastases by decreasing RhoC expression and consequently attenuating the transmigration and invasive ability of cancer cells. In fact, RhoC induces the sequential activation of Pyk2, FAK, MAPK and Akt pathways [199] that lead to the activation of MMP9. Further studies showed that miR-509 indirectly inhibits TNF-α, a cytokine that is also known to increase the permeability of the BBB [200]. Thus, it can be inferred that miR-509 suppresses brain metastases, not only by targeting RhoC but also by blocking TNF-α-induced BBB penetration.

Another miRNA related with BCBM worth mentioning is miR-181c that was shown to have an active role in BBB destruction during the process of formation of brain metastases [188]. It was further demonstrated that BCCs-derived extracellular vesicles promote brain metastasis development in vivo by increasing the permeability of the BBB through the disruption of TJs, whereas inhibition of extracellular vesicles secretion suppressed the invasiveness of malignant cells through the BBB. A mechanism involved in BBB disruption appears to be mediated by the extracellular vesicles’ release of miR-181c. MiR-181c promotes the downregulation of its target gene *PDPK1* and the consequent diminished expression of PDPK1 protein, leading to decreased phosphorylation of cofilin and resulting in activated cofilin-induced modulation of actin dynamics that disturbs BBB integrity [188]. Since the TJ proteins are in deep association with the actin cytoskeletal network, the disassembly of the actin cytoskeleton induces the relocation of these proteins, leading to the disruption of TJs and consequent increase of BBB permeability that facilitates the metastatic process [201].

MiR-122 has been previously identified as a marker for predicting metastatic progression in early stage BC by being highly expressed in BC patients’ serum prior to development of metastases [202]. More recently, it was demonstrated that miR-122 is also produced and secreted by BCCs to promote not only metastases in the lung, but also in the brain [184]. The authors found out that extracellular miR-122 downregulates the glycolytic enzyme pyruvate kinase in the pre-metastatic niche cells. This reduces glucose uptake by non-malignant cells and increases nutrient availability for cancer cells in the target organs [184]. Enhanced glucose uptake is a common feature in cancer due to the high energy demand in cancer cells and the low ATP-generating efficiency. Indeed, glycolytic enzymes have been shown to be upregulated in BC [203,204]. These findings suggest that circulating miR-122 can be a good biomarker for the early detection of BCBM and can modulate the microenvironment of brain metastases from BC.

Another stroma-derived extracellular miRNA, miR-19a, is capable of modulating BCBM microenvironment by downregulating PTEN [205], an important tumor suppressor that is frequently deleted in triple-negative BCBM patients and associated with poor prognosis [206]. In cases of BCBM, PTEN reduces the activation of Akt signaling pathway that has been shown to mediate the crosstalk between breast and glial cells in brain metastases leading to rapid disease progression [170]. As demonstrated by Zhang et al. [205], both human and mouse BCCs loose PTEN expression when disseminating in the brain, but not in other organs, and its expression is restored after the cells leave the microenvironment. The authors showed that the suppression of PTEN within the microenvironment is directly mediated by miR-19a, which is released by astrocytes. The decrease of PTEN led to an increase of chemokine C–C motif ligand 2 secretion and recruitment of myeloid cells that favor brain metastases of BC. This study is a good example of how miRNAs can influence the crosstalk between malignant cells and brain cells to create a microenvironment that is supportive to BCBM growth.

Finally, it was recently demonstrated the downregulation of miR-802-5p and of miR-194-5p in plasma prior to the detection of brain metastases, in a mouse model of preferential formation of brain metastases from BC [207]. Thus, miR-802-5p and of miR-194-5p appear as early biomarkers of BCBM, suggesting that their determination in liquid biopsies may constitute a new strategy for identification of BC patients at risk of developing brain metastases, or of those patients with micrometastases not suitable to be detected with currently available approaches. A bioinformatic and a bibliographic search revealed the transcription factor myocyte enhancer factor 2C (MEF2C) as a potential target of both miRNAs. Interestingly, analysis of the brain parenchyma revealed a progressive increase in the expression of MEF2C along metastases development and further showed the translocation into the nucleus of the transcription factor. Importantly, these observations coming out from the analysis of mouse brain sections were validated by analysis of resected brain metastases from BC patients. Hence, these results point to miR-802-5p and of miR-194-5p as tumor suppressors and to its target, MEF2C, as a new player in BCBM formation [207]. Another recent study revealed that miR-4428 and miR-4480 in serum samples could significantly distinguish BC patients with and without brain metastases, pointing to these miRNAs as predictive biomarkers of brain metastases in BC patients [208].

## 5. Conclusions and Future Perspectives

MiRNAs have arisen as important posttranscriptional regulators of possibly all the genes present in the human genome and overwhelming amounts of data have recently linked aberrant miRNA expression to the origin and development of many, if not all, types of cancer to which specific miRNAs signatures can be assigned. Moreover, some miRNAs have been specifically related to the formation of metastases and particularly from BC to the brain. However, the role of miRNAs in the cellular mechanisms and intercellular communication underlying cancer development and metastases progression remain mostly unmapped. Therefore, extending the current knowledge about miRNAs biopathology will pave the way for the establishment of miRNAs as early biomarkers and as potential targets for modulation. Hopefully, the anticipated discoveries in the years to come will open new opportunities for a timely detection and therapeutic intervention, essential to improve the expectancies in the field of oncology and particularly of neuro-oncology.

## Figures and Tables

**Table 1:** MiRNAs associated with breast cancer brain metastases and their expression in cancer cells.

MiRNA	Type of Study	Breast Cancer Cell Lines	Expression in Brain Metastases vs. Primary Tumor	Expression in Metastatic Tumors vs. Nonmetastatic Tumors	Putative Targets	Ref.
miR-7	In vitro and in vivo	MDA-MB-231 and MCF-7	Downregulated	–	KLF4	[180]
miR-10b	In vitro	MDA-MB-231 and MDA-MB-468	Upregulated	Upregulated	HOXD10 and MICB	[181]
miR-19a	In vitro and in vivo	MDA-MB-231BR	–	Downregulated	Unknown	[182]
miR-20b	In vitro and in vivo	MCF-7 and MDA-MB-231	Upregulated	Upregulated	PTEN	[183]
miR-29	In vitro and in vivo	MDA-MB-231BR	–	Downregulated	Unknown	[182]
miR-122	In vitro and in vivo	MDA-MB-231-HM	Upregulated	–	PKM	[184]
miR-141	In vivo	SUM149, MDA-MB-231BR and MDA-IBC3	Upregulated	Upregulated	Unknown	[185]
miR-210	In vitro and in vivo	MDA-MB-231BR	–	Upregulated	Unknown	[182]
miR-509	In vitro and in vivo	MDA-MB-231 and MCF-7	Downregulated	Downregulated	RhoC and TNF-α	[186]
miR-524-5p	In vitro and in vivo	MCF-7 and MDA-MB-231	Downregulated	–	BRI3, ERK pathway	[187]
miR-181c	In vitro and in vivo	MDA-MB-231	Upregulated	–	Cofilin	[188]
miR-1258	In vitro and in vivo	MDA-MB-231BR1 and MDA-MB-231BR3	Downregulated	Downregulated	Heparanase	[189]
